# Development and evaluation of a bead-based Multiplexed Fluorescent ImmunoAssay (MFIA) for detection of antibodies to *Salmonella enterica* serogroup B and C_1_ in pigs

**DOI:** 10.1186/s12917-022-03362-w

**Published:** 2022-07-05

**Authors:** Sanne Schou Berger, Ulla Riber, Vibeke Frøkjær Jensen, Klara Tølbøll Lauritsen, Lars Ole Andresen

**Affiliations:** 1 Centre for Diagnostics, DTU Health Technology, Technical University of Denmark, 2800 Kgs. Lyngby, Denmark; 2 National Veterinary Institute, Technical University of Denmark, 2800 Kgs. Lyngby, Denmark

**Keywords:** Salmonella, Antibodies, Serology, Lipopolysaccharide, Luminex, Immunoassay

## Abstract

**Background:**

Since 1995, a surveillance program for *Salmonella* has been applied in the Danish pig industry in order to reduce cases of human salmonellosis. The objective of this study was to develop a bead-based Multiplexed Fluorometric ImmunoAssay (MFIA) as an improved serological surveillance method compared to the *Salmonella* mix ELISA, which has been the national reference immunoassay in the Danish *Salmonella* surveillance program for about 20 years.

**Results:**

An MFIA for detection of antibodies to *Salmonella* serogroup B and C_1_ was developed and optimized with regard to coupling of beads with *Salmonella* lipopolysaccharide antigens and establishing suitable assay conditions. The *Salmonella* MFIA was validated by testing sera from experimentally infected pigs as well as field sera from non-infected and infected pig herds, and by comparing to results from the *Salmonella* mix ELISA, which was run in parallel. Sensitivity and specificity was evaluated using receiver operating curve analysis showing an area under curve for the serogroup B and C_1_ MFIA of 0.984 and 0.998, respectively. The *Salmonella* MFIA was shown to detect more antibody-positive samples in seropositive herds compared to the *Salmonella* mix ELISA, and Bayesian statistics confirmed that the MFIA had a considerably higher sensitivity (94.5%) compared to the mix ELISA (75.1%). The assay specificity was slightly lower for the *Salmonella* MFIA (96.8%) compared to *Salmonella* mix ELISA (99.5%). Coupled beads were stable for at least 1 year at 4˚C, and MFIA reproducibility and repeatability of the *Salmonella* MFIA were acceptable. Results from proficiency tests also indicated that the *Salmonella* MFIA was more sensitive than the *Salmonella* mix ELISA and that they had similar specificity.

**Conclusions:**

A bead-based MFIA for simultaneous detection of porcine serum antibodies to *Salmonella enterica* serogroup B and C_1_ was developed and implemented in the Danish porcine serological *Salmonella* surveillance program in 2018. The *Salmonella* MFIA can distinguish, as opposed to the *Salmonella* mix ELISA, between antibodies to serogroup B and C_1_ and the MFIA shows considerably better sensitivity.

**Supplementary Information:**

The online version contains supplementary material available at 10.1186/s12917-022-03362-w.

## Background

Infections with bacteria belonging to the species *Salmonella enterica* occasionally cause clinical disease in pigs, but infected pigs also pose a threat to human health since they form a major zoonotic reservoir [1]. Especially *Salmonella enterica* serovar Typhimurium and serovar Infantis are known to be important and prevalent pathogens that induce risk of severe infection and complications in human consumers. *S. enterica* subspecies are divided into serogroups based on similarities in the composition of their cell wall expressed LPS (O antigens) and flagellar proteins (H antigens), as described in the White-Kauffman-Le Minor scheme [2].

In order to reduce human cases of salmonellosis, a surveillance program for *Salmonella* has been applied in the Danish pork industry since 1995 [3]. As part of the surveillance program, an Enzyme-Linked Immunosorbent Assay (ELISA) designated *Salmonella* mix ELISA was employed to screen porcine serum samples and meat juice for antibodies to *Salmonella* serogroup B and C_1_. The mix ELISA is based on the use of purified lipopolysaccharides (LPS) for detection of *Salmonella* serogroup specific antibodies. It was developed at the Danish National Veterinary laboratory and has since then been maintained in the laboratory as the national reference ELISA in the Danish porcine *Salmonella* surveillance program [4].

The LPS employed in *Salmonella* mix ELISA contains serogroup B specific O:1,4,5,12 antigens from *S*. Typhimurium, and serogroup C_1_ specific O:6,7 antigens from *S*. Choleraesuis that are identical to *S*. Infantis O-antigens [3]. Hence, *Salmonella* mix ELISA also detects antibodies against the remaining serovars included in the B and C_1_ serogroups. Due to the assay design of the *Salmonella* mix ELISA, it does not distinguish between antibodies to serogroup B and C_1_.

For decades, in-house ELISAs have also been used for diagnosis and surveillance of other diseases than salmonellosis in Danish pig herds, including those caused by Porcine Reproductive and Respiratory Syndrome Virus (PRRSV) and the bacterium *Actinobacillus pleuropneumoniae* [5–8]. A single serum sample often has to be tested for antibodies to all of these pathogens, but testing serum samples in separate ELISAs is resource demanding and time-consuming. In order to optimize this, a serological Multiplexed Fluorometric ImmunoAssay (MFIA) was developed and implemented based on the commercial xMAP technology platform developed by Luminex Corp., which can detect antibodies to multiple pathogens simultaneously. This assay setup utilizes magnetic polystyrene beads containing a combination of two fluorescent dyes that differentiate the beads into regions. Beads from these various regions are coated with antigens from different pathogens, making it possible to detect bound serum antibody with layers of biotinylated anti-porcine IgG and a fluorescent streptavidin-R-phycoerythrin (R-PE) reporter conjugate. The interaction between beads and sample antibodies is measured by flow cytometry, whereby the antigen is identified by the internal bead fluorescence and the level of bound sample antibodies is measured by intensity of the R-PE reporter.

In contrast to the *Salmonella* mix ELISA, a two-plex *Salmonella* MFIA offers the possibility of distinguishing between antibodies to *Salmonella* serogroup B and C_1_. It also facilitates simultaneous detection of serum antibodies to other pathogens important in pig production, such as *A. pleuropneumonia* and PRRSV. A serological MFIA that detects and distinguishes between antibodies to seven serovars of *A. pleuropneumonia* in pigs has previously been developed, implemented, and described [9, 10]. The additional serological MFIA that differentiate antibodies to PRRSV type 1 and PRRSV type 2 has recently been developed and implemented (manuscript in preparation).

Herein, we describe the development and validation of a bead-based multiplexed immunoassay that detects and distinguishes between antibodies against *Salmonella* serogroup B and C_1_ within a single serum sample volume.

## Results

### Assay optimization and investigation of reagent stability

Common assay conditions were identified for the *Salmonella* serogroup B and C_1_ MFIAs based on optimal signal-to-noise ratios. The same assay conditions were applicable to MFIAs for *A. pleuropneumoniae* [9, 10] and PRRSV (manuscript in preparation).

**Fig. 1:**
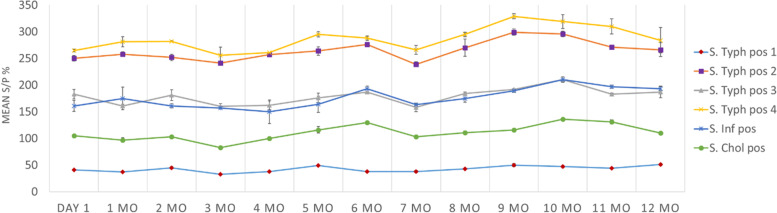
Time-dependent antibody-binding activity of beads coupled with LPS from *Salmonella* serogroup B and C_1_. Antigen-coupled beads were tested with the Quality Control (QC) panel after 1 day and then monthly for one year. Binding of antibody to beads was calculated as an S/P% value. The plot shows mean S/P% values ± STD for each of the four serogroup B positive sera (*S*. Typhimurium) and the two serogroup C_1_ sera (*S*. Infantis and *S*. Choleraesuis)

In a shelf life study, antigen-coupled beads maintained a stable interaction with serum antibodies for 13 months when stored at 4˚C (Fig. 1). Figure 1 shows the time-dependent binding activity of LPS-coupled beads to antibodies from each of the four serogroup B positive sera and the two serogroup C_1_ positive sera. The antibody-binding activity is expressed as the mean percent sample-to-positive ratios (S/P% values)). The results of the shelf life studies were used for estimating the between-run repeatability of the *Salmonella* MFIA. The percent coefficient of variance (CV%) of the calculated S/P% values were acceptable (< 15%) for each of the four serogroup B sera (7.0%, 7.8%, 8.5%, 13.3%) and for the two serogroup C_1_ sera (10.6%, 13.5%).

Within-run repeatability (within one plate) showed acceptable CV% values (i.e. below 15%) of 2.0%, 3.9%, 4.9% and 13.3% for the four serogroup B positive sera and 4.0% and 5.9% for the two serogroup C_1_ positive sera.

To document linearity in the MFIA, serum samples with S/P% > 300 were diluted in negative serum. Testing serial dilutions of such samples, showed linearity (Supplementary Fig. 1).

### MFIA validation

**Fig. 2:**
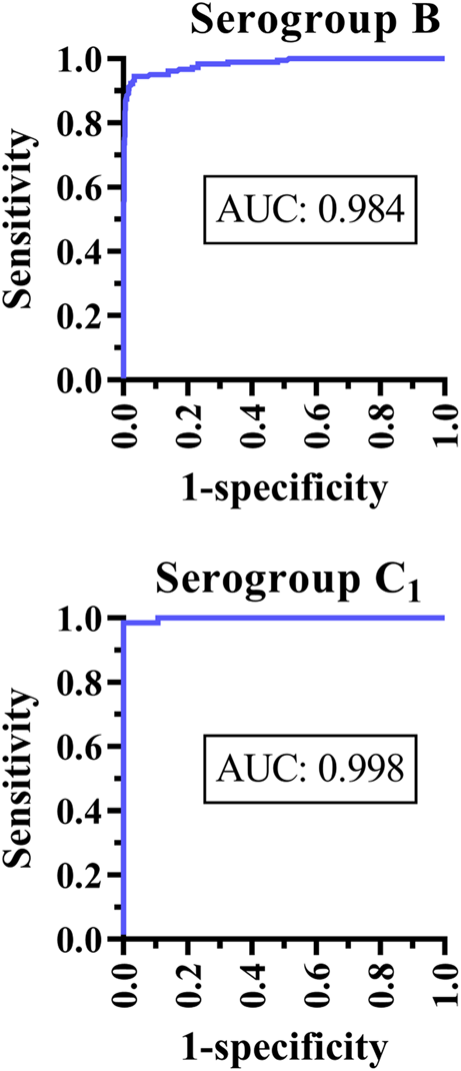
Receiver Operating Curves (ROCs) for *Salmonella* serogroup B and C_1_ multiplex-analysis with Area Under Curve (AUC) shown in each graph. The reference assay was an in-house *Salmonella* mix ELISA

Receiver Operating Curve (ROC) analysis was performed for *Salmonella* serogroup B MFIA using results from the validation with serum samples from naturally infected and non-infected pig herds. Results of the serogroup B MFIA were comparable to those of the mix ELISA with an area under the curve (AUC) of 0.984 (Fig. 2).

The cut-off for the serogroup B MFIA was six at the optimal differential positive rate (DPR), and the sensitivity and specificity at this cut-off was 96.6% and 94.5% respectively (Table 1).

**Table 1:** Sensitivity, specificity and cut-off values for the *Salmonella* serogroup B and C_1_ MFIA

	**Serogroup B**	**Serogroup C**_**1**_
**Optimal DPR**^a^	0.912	0.985
Cut-off	6	5
Specificity (%)^b^	96.6 (95.0–97.7)	100.0 (94.5–100.0)
Sensitivity (%)^b^	94.5 (90.2–97.4)	98.5 (92.0–100.0)
**Adjusted cut-off values**^c^		
DPR	0.896	0.940
Cut-off	10	10
Specificity (%)^b^	97.8 (96.5–98.7)	100.0 (94.5–100.0)
Sensitivity (%)^b^	91.8 (86.8–95.3)	94.0 (85.4–98.4)

^a^Statistical values calculated at the optimal Differential Positive Rate (DPR). ^b^95% confidence interval values are shown in parentheses. ^c^Values calculated with cut-offs adjusted to 10

The serogroup C_1_ MFIA was also tested for reactivity with the 1425 samples in a duplex together with the serogroup B MFIA, but only results with samples from experimentally infected pigs were used for ROC curve analysis, since the prevalence of serogroup C_1_ was very low in the tested herds. ROC curve analysis for the serogroup C_1_ showed comparable results to mix ELISA with an AUC of 0.998 (Fig. 2). At the optimal DPR, the cut-off for this assay was five with a sensitivity of 100% and a specificity of 98.5% (Table 1).

For future use of the *Salmonella* MFIA in Danish swine herds, the cut-off values were adjusted to 10 in order to minimize the number of false positive reactors. Table 1 shows the specificities, sensitivities and DPR after cut-off adjustment.

**Fig. 3:**
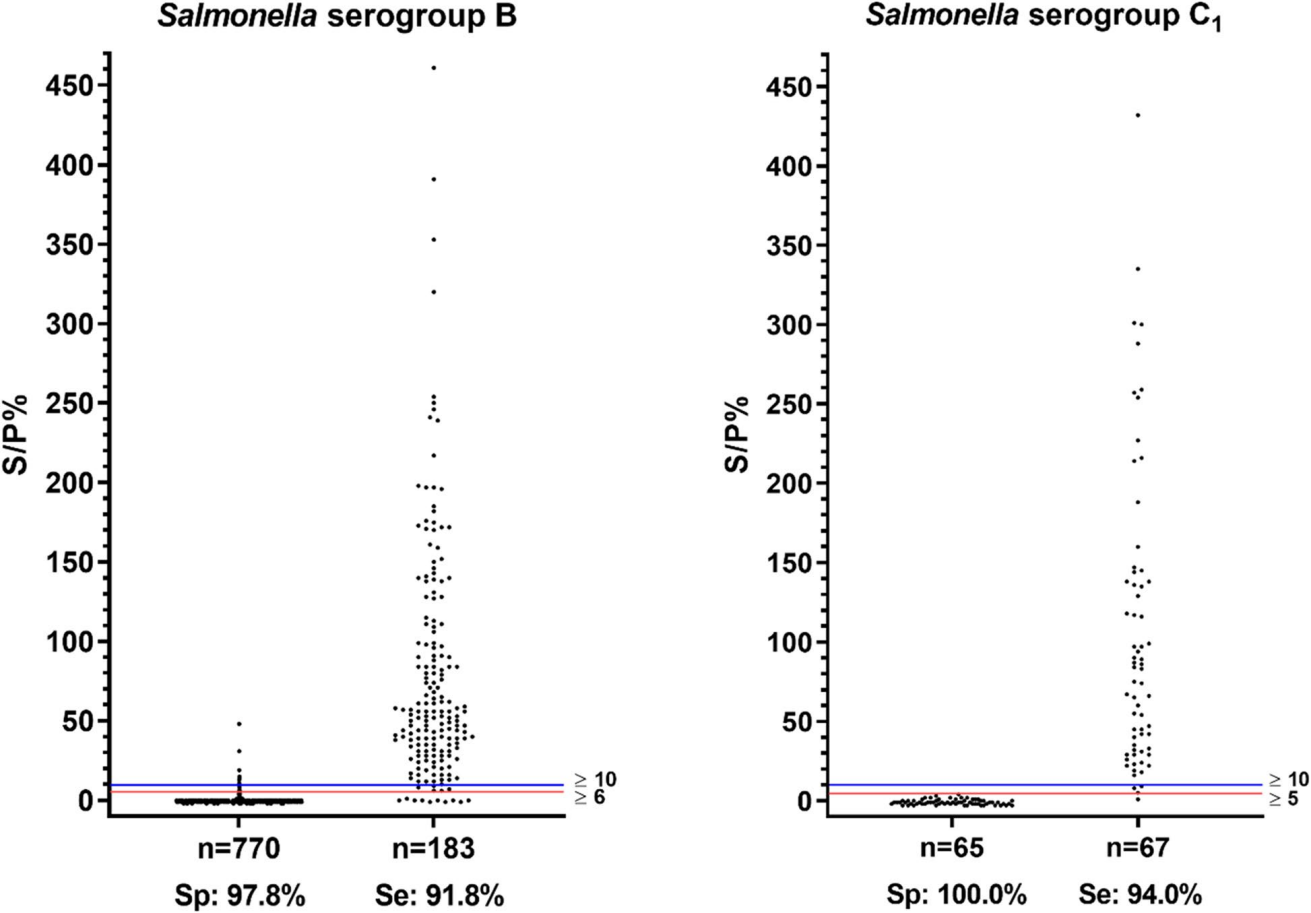
Dot plots with results from the validation of the *Salmonella* serogroup B and C_1_ MFIA. Reactivity with antibodies in serum samples from *Salmonella* mix ELISA-negative pigs (left) and *Salmonella* mix ELISA-positive pigs (right). Red horizontal lines indicate the cut-off values at the optimal Differential Positive Rate and blue horizontal lines show adjusted cut-off values. Below each graph are shown specificities (Sp) and sensitivities (Se) at the specified cut-off. Levels of antibodies in serum samples is expressed as a percent sample-to-positive ratio S/P%

Sensitivities, specificities and cut-off values are also indicated in a dot plot (Fig. 3), which shows reactivity in *Salmonella* MFIA with antibodies in serum samples that had negative or positive reaction in the *Salmonella* mix ELISA.

**Fig. 4:**
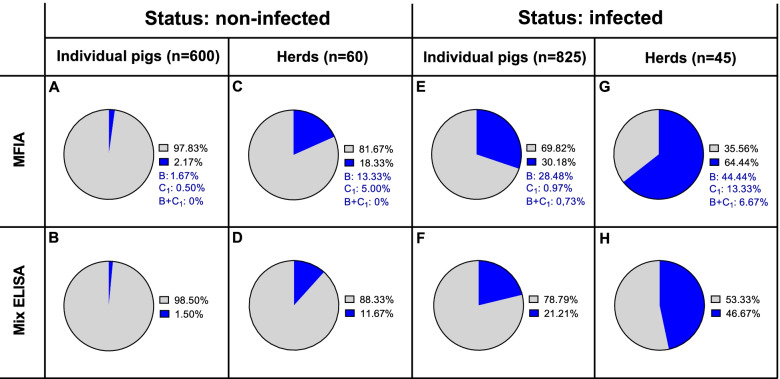
Percentages of 1425 swine samples from 105 herds that tested negative (grey) or positive (blue) in MFIA and mix ELISA for antibodies to *Salmonella* enterica serogroup B and C_1_ in herds previously categorized with a status as *Salmonella* free (**A**-**D**) or *Salmonella* infected (**E**–**H**). Since MFIA distinguishes between serogroup B and C_1_, percentage of pigs or herds infected with the different serogroups are indicated with blue text

Figure 4 shows pie charts that compare the results (using a cut-off of 10 for MFIA) of the 1425 samples tested in MFIA and mix ELISA at individual and herd level in relation to the herd status (non-infected or infected with *Salmonella*). In “non-infected” herds, MFIA detects 18.33% and mix ELISA detects 11.67% positive herds (Fig. 4C, D), while in “infected” herds, MFIA detects 64.44% positive herds and mix ELISA detects 46.67% positive herds (Fig. 4G, H). With MFIA, ~ 30% additional herds were therefore classified as positive compared to results obtained with mix ELISA.

If hypothesizing that samples which are negative in mix ELISA are true negatives, these would according to Table 2 represent false positive reactions when positive in MFIA. The relative risk of positive samples in MFIA, given a negative mix ELISA result, was 11.38% for samples from the herds classified as infected and 0.68% for samples from the herds classified as non-infected (Table 2). There is a significant difference of the risk depending on herd class (Fishers exact test: p < 0.001, with a relative risk of 16.82). If hypothesizing that samples which are negative in mix ELISA are true negatives, these would according to Table 2 represent false positive reactions when positive in MFIA. If the hypothesis were true, we would have expected that the risk of an MFIA positive result for a mix ELISA negative sample would be similar whether the true negative sample was from herds classified as non-infected or infected. Since there is a highly significant difference of the risk depending on herd class (Table 2) the hypothesis can therefore be rejected.

**Table 2:** The risk of false positive results in MFIA in herds classified as *Salmonella-*infected and non-infected

		**MFIA result for pigs that are negative in mix ELISA**		**Total**	**Risk of MFIA “false” positive results (%)**
		*Salmonella* positive pigs in MFIA	*Salmonella* negative pigs in MFIA		
**Herd classification**	Infected	74	576	650	11.38
	Non-infected	4	587	591	0.68

Data comprise only pigs classified as negative based on mix ELISA. The relative risk (11.38/0.68) is 16.82

A Hui-Walter test performed on the tested herds, divided into two populations based on a “red/blue” classification system integrated into the Danish Specific Pathogen Free (SPF) system [11, 12], showed that MFIA had a sensitivity of 94.5% compared to mix ELISA with a sensitivity of 75.1%. Assay specificity 96.8% for MFIA and 99.5% for mix ELISA (Table 3).

**Table 3:** Estimated sensitivities and specificities for MFIA and mix ELISA using Bayesian statistics (Hui-Walter test)

Parameter	Diagnostic test	Median (95% CI)
*Salmonella* prevalence in production herd populations		42.1
*Salmonella* prevalence in breeding herd populations		4.2
Sensitivity (%)	MFIA	94.5
	Mix ELISA	75.1
Specificity (%)	MFIA	96.8
	Mix ELISA	99.5

Proficiency test results provided by GD Deventer from year 2015 to 2020 (8 samples per proficiency test), showed that MFIA correctly identified more samples than mix ELISA that were positive for antibodies to *S. enterica* serovar Brandenburg (serogroup B). In contrast to MFIA, mix ELISA did not detect samples that were positive to *S. enterica* serovar Livingstone (serogroup C_1_). The antigens included in MFIA and mix ELISA are not specific for serogroup C_2_ and serogroup D_1_. Therefore, *S. enterica* serovar Goldcoast (C_2_) antibody positive samples were not detected positive in any of the proficiency tests, while it alternated whether the *S. enterica* serovar Panama (D_1_) antibody positive samples tested positive in both assays. The latter finding is caused by cross-reactivity, which is observed between antibodies to serogroup B and D_1_ LPS antigens due to shared O-antigens (Table 4).

**Table 4:** Proficiency test results for samples tested from 2015 to 2020 in MFIA and Mix ELISA

				**Number of proficiency test samples correctly identified as negative or positive**^a^		
***Salmonella enterica* serovar**	**Serogroup**	**O-antigen**	**# samples tested**	**MFIA serogroup B**	**MFIA serogroup C**_**1**_	**Mix ELISA** **Serogroup B + C**_**1**_
Negative	-	-	11	11	11	11
Brandenburg	B	4,[5],12	8	8	0	4
Typhimurium	B	1,4,[5],12	14	14	0	14
Livingstone	C_1_	6,7,14	6	0	6	0
Goldcoast^b^	C_2_	6,8	3	0	0	0
Panama^c^	D_1_	1,9,12	6	3	0	3

^a^Sample ID is unknown to proficiency test participants, so samples may reoccur from year to year

^b^The O-antigens used in MFIA and mix ELISA are not 6,8

^c^Since the D_1_ O-antigen is not included in the test, the reactants observed are due to cross-reaction with the serogroup B O-antigen

## Discussion

When the *Salmonella* mix ELISA was included in the surveillance program in the mid-1990’ies, the intention of the Danish authorities was to use it as a serological screening tool that could supplement the less sensitive bacteriological cultivation methods [3]. It is important for a screening tool to have a high sensitivity and although the mix ELISA was a good solution at the time, more refined alternatives have since emerged, such as MFIAs represented by the Luminex xMAP Technology. MFIAs are known to include sensitive assays with high dynamic ranges [13–16].

In order to determine sensitivity and specificity of the developed *Salmonella* MFIA we used ROC curve statistical analysis, where the MFIA was compared to the *Salmonella* mix ELISA. However, comparison of a new assay with a reference assay would result in false positives or negatives with the new test that are indeed true positives or negatives, if the new assay is more sensitive or specific than the reference assay. Consequently, the sensitivity and/or specificity of the new test would be underestimated. In ROC curve analysis, results were analyzed at the individual level. The same results were also analyzed at herd level, where we compared the risk of a positive sample in MFIA, given a negative mix ELISA result, in herds previously classified as either infected or non-infected. There was a highly significant relative risk depending on herd class indicating that a large proportion of the MFIA positive samples with a negative mix ELISA result, were most likely true positives (Fig. 4, Table 2). This suggests that MFIA data are more reliable than mix ELISA data for detecting positive herds.

In addition to using ROC curve analysis we also estimated sensitivity and specificity of the MFIA using the Hui-Walter paradigm, which is based on Bayesian statistics [17]. This paradigm is used for estimating sensitivity and specificity of two tests, when a definite “gold standard” reference assay is not available. The Hui-Walter test was performed on datasets from herds divided into two populations based on a “red/blue” classification system integrated into the Danish Specific Pathogen Free (SPF) system [11, 12], where *Salmonella* prevalence is much higher in production herds (“blue” populations) than in breeding herds (“red” populations). The Hui-Walter test showed a higher sensitivity of 94.5% for the MFIA compared to a sensitivity of 75.1% for the mix ELISA. The assay specificity was slightly higher for mix ELISA (99.5%) compared to MFIA (96.8%) (Table 3). Hereby it was shown that, despite using the same LPS antigen preparations for detection of antibodies, the developed MFIA is more sensitive than mix ELISA, detecting more *Salmonella* antibody-positive samples in seropositive herds.

By facilitating testing for and differentiation of multiple targets, Luminex technology is suitable for screening populations for antibodies to multiple targets using a single sample volume. In the *Salmonella* mix ELISA, two LPS antigens (serogroup B and C_1_ specific) are coupled to the bottom of the same wells, which may introduce an interference so that antigens are not presented optimally or equally. This interference is not an issue in bead-based MFIA, since different subsets of beads are coupled with different antigens. Thus, a higher sensitivity of the *Salmonella* MFIA compared to *Salmonella* mix ELISA probably also originate from a more optimal antigen presentation on the surface of beads. Importantly, the presentation of *Salmonella* antigens on separate bead subsets in the MFIA provides a major diagnostic advantage compared to the *Salmonella* mix ELISA, since the MFIA allows distinction between antibodies to serogroup B and C_1_.

The cut-off values identified using ROC curve analysis for *Salmonella* serogroup B and C_1_ MFIAs were adjusted to 10 to increase specificity, in order to reduce the probability of getting false positive results. False positive results in the Danish surveillance system can have severe and unnecessary consequences for the individual swine farm, including trading restrictions and demands for resampling and retesting. Since the tested sample size from each herd is normally 10 (or even higher when sampling for antibody profiles in a pig herd), and given that the sensitivity is > 90% even after increasing the specificity, the surveillance is still highly sensitive with the new cut-off, with regards to detecting *Salmonella* antibodies in truly positive herds.

The measurement of R-PE fluorescence intensity as a measure of sample antibody levels in the MFIA has a large linear range, although the validated *Salmonella* positive reference (S/P% = 100), only uses around 35–40% of the estimated maximal signal. Therefore, highly positive samples may be calculated 3–4 times higher than the positive reference sample. Testing serial dilutions of *Salmonella* antibody positive serum showed linearity (Supplementary Fig. 1), which makes it possible to report samples with high levels of antibody directly, without pre-dilution. In contrast, ELISAs generally have a maximum optical density and a limited range of linearity, which may require pre-dilution of samples to determine “highly positive” samples correctly.

The *Salmonella* MFIA has been employed in the Danish surveillance program since 2018. Identical assay conditions are used in all our in-house developed MFIAs for porcine serum samples, which permit simultaneous analysis for *Salmonella* serogroup B and C_1_, *Actinobacillus pleuropneumoniae* serovar 1, 2, 5, 6, 7, 10 and 12 [9, 10] and PRRSV type 1 and 2 (manuscript in preparation). In addition, a subset of the MFIAs can be run according to customer requests. The detection of antibodies to multiple analytes within a serum sample reduces the required amount of serum sample, as well as the amount of time and labor needed to test the samples. This shortens the response time and significantly lowers the costs for serological testing and surveillance.

Future MFIAs for other targets could include *Salmonella* assays for detection of antibodies in porcine sera to serogroups other than B and C_1_ that include serovars with zoonotic potential. Furthermore, there may be a potential to include serological testing for other important pathogens in the pig production.

## Conclusion

In conclusion, the serological bead-based MFIA described here for detection of antibodies to *Salmonella* serovars belonging to serogroup B or C_1_ has a good repeatability and a high sensitivity and specificity compared to the alternative; the *Salmonella* mix ELISA.

## Methods

### Serum samples

For assay optimizations, testing assay repeatability as well as the shelf life of coupled beads, a serum panel was used. This panel contained four sera positive for antibodies to *Salmonella* serogroup B (separate sera with different levels of antibodies to *S*. Typhimurium), two sera positive for antibodies to *Salmonella* serogroup C_1_ (one with antibodies to *S*. Cholerasuis and one with antibodies to *S*. Infantis), as well as a *Salmonella* negative serum pool. These sera originated from experimental *Salmonella* infection studies performed decades ago at the National Veterinary Laboratory, Technical University of Denmark, and for years used as part of a QC serum panel for routine evaluation of the performance of ELISAs measuring *Salmonella* antibodies to serogroup B and C_1_ [4].

For assay validation, two samples from the serum panel that were positive for antibodies to either serogroup B and C_1_ were pooled, and this serum pool was used as a combined positive control sample applied to each assay plate during the validation procedure along with serum from a *Salmonella* antibody-negative pig.

Field samples used for the final validation of the *Salmonella* MFIA included 1425 samples collected in 2016 from 105 Danish pig herds participating in the *Salmonella* surveillance program with routine ELISA screening, as well as samples tested for serological diagnosis of *Salmonella* infection in pig herds. The herd status was according to the classification at the time of blood sampling that is reported and updated monthly on the herd health status homepage of the SPF Health, Danish Agriculture and Food Council [11]. Classification was established by serological surveillance and culture from faecal and environmental samples. Samples from herds classified as infected had at some point prior to the time of sampling for the present study been either serologically positive for antibodies to *Salmonella* or positive for *Salmonella* by bacteriological examination. If a herd on just one occasion during the surveillance programme had serologically *Salmonella*-positive results in sample sizes of ten samples per examination or was positive for *Salmonella* by culture this would classify the herd as infected. Hence, infected herds could have a true positive rate varying between 0 and 100%. It can be assumed that the non-infected herds had a true positive rate of 0%.

The overall herd health status is classified into safety levels designated red and blue, where red herds have higher biosecurity and health control levels than blue herds, which means that the frequency of *Salmonella* infection is lower in red herds than in blue herds [11].

Since *Salmonella* serovars belonging to serogroup C_1_ are rare in Denmark, serum samples, obtained from two previous studies with experimentally infected pigs, performed at the National Veterinary laboratory, were used for the validation. One study was from 1998/1999 with 12 pigs inoculated with *S. enterica* serovar Infantis, and another study from 2000/2001 with six pigs inoculated with *S. enterica* serovar Choleraesuis (both unpublished).

Proficiency tests for serological porcine *Salmonella* testing were provided by GD Deventer, the Netherlands from the year 2015 to 2020 and included eight freeze-dried serum samples per year.

### Preparation of lipopolysaccharide

Lipopolysaccharide (LPS) antigen from *Salmonella enterica* serovar Typhimurium strain no. 3389–1/92 and *Salmonella enterica* serovar Choleraesuis var. Kunzendorf strain no. 143 were prepared after cultivation in a bioreactor and purified by extraction with hot phenol as previously described [18]. These LPS antigen preparations are also used as detection antigens in the *Salmonella* mix ELISA.

### Coupling of lipopolysaccharide to magnetic beads

LPS preparations from *Salmonella* serovars belonging to serogroup B (LPS from *S*. Typhimurium) and C_1_ (LPS from *S*. Cholerasuis) were coupled to separate subsets of magnetic beads (Bio-Plex Pro magnetic COOH beads, Luminex) using a method described previously [9, 19]. Briefly, 1.25 × 10^7^ beads were resuspended in 250 μL of 0.1 M 2-(*N*-morpholino)ethanesulfonic acid (MES buffer; pH 5.0; M 22,933, Sigma-Aldrich, St. Louis, MO) and then vortexed (10 s) and sonicated (20 s; Sonorex Digitec, Bandelin, Berlin, Germany). A volume of 750 μL LPS diluted in MES buffer and 25 μL of fresh 1-ethyl-3-(3-dimethylaminopropyl)carbodiimide hydrochloride (EDC; PG82079, Thermo Fisher Scientific) solution (50 mg/mL) was added. After vortexing, the solution was incubated for 40 min in the dark at room temperature (RT) in a rotator (PTR-35, Grant Instruments, Cambridge, England). An additional 1 mL of LPS, together with 25 μL fresh EDC, was added and incubated with the beads for 40 min in the dark at RT in the rotator. The last incubation step was repeated for a total of 3 incubations with LPS. A magnet (DynaMag-5, Thermo Fisher Scientific, Waltham, MA) for 5-mL tubes (Eppendorf Protein LoBind tube, Sigma-Aldrich) was used for washing the beads (3 times) with 3 mL of phosphate-buffered saline (PBS), 0.1% bovine serum albumin (BSA), 0.02% Tween 20, and 0.05% sodium azide (PBS-TBN; pH 7.4). The beads were stored in the dark at 2–8 °C in 1 mL of PBS-TBN. The coupling procedures were optimized with regard to amount of antigen, buffers, incubation time as well as conditions providing the highest signal-to-noise ratio of the median fluorescent intensities (MFIs) measured after testing the beads with serum samples in the process of assay development.

### Multiplexed Fluorometric ImmunoAssay (MFIA)

Suspensions of beads coupled with LPS from serogroup B and C_1_, respectively, were vortexed (10 s) and mixed at a concentration of 8 × 10^4^ beads/mL per bead subset in a single volume of assay buffer A (PBS 0.05 M, 0.05% Tween, 1% BSA, 0.5 M NaCl). After vortexing (10 s) and sonication (20 s), 25 μL of bead suspension was added to black flat-bottom, 96-well plates (Bio-Plex Pro, Bio-Rad, Hercules, CA) together with 25 μL of pig serum diluted in assay buffer A (final serum dilution, 1:200). All incubations were performed at RT in the dark (covered with aluminum foil) on a rotating shaker (MTS 2/4 digital microtiter shaker, IKA-Werke, Staufen, Germany). The plates were incubated for 60 min and washed with wash buffer (PBS, 0.05% Tween) using an automated plate washer for magnetic beads (ELx405, BioTek, Winooski, VT). Next, 25 μL biotin-conjugated rabbit anti-swine IgG (SAB3700429, Sigma-Aldrich) was added per well (2 μg/mL, diluted in assay buffer B (PBS, 0.05% Tween, 1% BSA), and incubated with the beads for 30 min. The plates were washed in the automated plate washer, and incubated for 30 min with 75 μL (1 μg/ml, diluted in assay buffer B) of Streptavidin–R-Phycoerythrin (S21388, Thermo Fisher Scientific). The plates were shaken on the rotating shaker for 30 s, and samples were read in a Bio-Plex 200 flow cytometric platform (Bio-Rad) adjusted to acquire a 50-μL sample and count a minimum of 50 beads/analyte. During validation, all samples were analyzed in duplicates. Bound pig serum IgG was measured as the MFI signal of R-phycoerythrin (R-PE) after exclusion of aggregated beads by gating. Data were acquired using the flow cytometric platform software (Bio-Plex Manager software v.6.1, Bio-Rad).

### Assay optimization and test of reagent stability

Parameters including assay reaction time, washing steps, buffer composition, incubation temperature as well as antigen-, serum-, and conjugate concentrations were optimized in single-plex assays using serum samples in the QC panel.

To evaluate the shelf life of coupled beads over a 13-month period duplicates of the samples included in the QC serum panel were tested monthly by two different operators (Fig. 1). Results of the shelf life validation studies were also used for measuring between-run repeatability of the analysis. A within-run repeatability study was performed with 12 separate dilutions of the samples from the QC serum panel that were run on the same assay plate. Assay repeatability was defined as the percent mean coefficient of variation of the MFI values (CV%).

### Data analysis

Percent sample-to-positive ratios (S/P%) were calculated using the following formula:

$$\mathrm S/\mathrm P\%=\frac{\left({\mathrm{MFI}}_{\mathrm{sample}}-{\mathrm{MFI}}_{\mathrm{negative\;control}}\right)}{\left({\mathrm{MFI}}_{\mathrm{positive\;control}}-{\mathrm{MFI}}_{\mathrm{negative\;control}}\right)}\times100$$

Receiver operating characteristic (ROC) curve analysis was used for the determination of test analysis quality compared to the existing in-house mix ELISA. Differential positive rates (DPR) (defined as sensitivity + specificity—1) were used together with dot plot correlation curves and 2*2 contingency tables to determine cut-off values for the individual analyte in the MFIA [20–22].

The statistical programs R and OpenBUGS were used for the Hui-Walter test.

### Assay validation

After optimization of the *Salmonella* multiplex assay, beads coupled with serogroup B and C_1_ LPS antigens were mixed and tested in a two-plex assay against 1425 field serum samples from 105 SPF herds. The same samples were tested concurrently in *Salmonella* mix ELISA [3] which is the national reference assay for surveillance of *Salmonella* in pigs and for herd classification in the Danish SPF system [11, 12].

#### Receiver operating curve analysis

In ROC curve statistical analysis, if a herd was found positive in the *Salmonella* mix ELISA, MFIA data were included for the positive sera while samples in the same herd that tested negative in the *Salmonella* mix ELISA were excluded in order to minimize the influence of animals undergoing seroconversion, which would give borderline reactions. Therefore, of the 1425 tested serum samples only 953 were included in the ROC curve analysis.

Since *Salmonella* serovars belonging to serogroup C_1_ are rare in Denmark, ROC-curve analysis for this serogroup was performed on results obtained by testing samples from naïve and experimentally infected pigs originating from two studies with samples before inoculation, and samples taken with regular intervals after inoculation. Samples collected during seroconversion (day 4–7 post inoculation) were not included in ROC curve analysis since these can show borderline reactions. Samples taken before seroconversion (before inoculation and day 1–3 post inoculation) were used as negative samples (n = 65), while samples taken after seroconversion were used as positive samples (n = 67).

#### Bayesian statistics

The Hui-Walter paradigm, which is based on Bayesian statistics can be used to estimate sensitivity and specificity in the absence of a definite “gold standard” [17]. The test is based on data from both assays applied to two (sub) populations with different prevalences. Here, we used two populations defined in the Danish SPF system based on different biosecurity measures and surveillance of specific porcine infectious diseases [11, 12]. Whereas the “blue” herds are mainly production herds with a high level of biosecurity, the “red” herds are breeding herds with the highest level of biosecurity. Division into herds with a status of ‘infected’ or ‘non-infected’ in the *Salmonella* surveillance system was not applied for the Hui-Walter test, because it would have introduced a statistical bias since it is based on results from the *Salmonella* mix ELISA [4].

## Supplementary Information

**Additional file 1:** **Supplementary Fig. 1. **Test of linearity in MFIA when titrating *Salmonella* serogroup B positive serum samples. Four serogroup B positive sera (A-D) with high calculated S/P% values were serially diluted in negative serum. The serum dilutions as well as the negative serum were analysed on two separate days. Figure A-D show mean median fluorescent intensities (MFI) ± STD for serogroup B responses, as well as a linear trend line and R^2^ for the linearity. The higher the MFI, the more specific antibodies are present in the sample. % serum sample represents the percentage of positive sample in negative serum.

## Data Availability

The datasets generated and/or analyzed during the current study are not publicly available due to the fact that swine herds may be identified from the datasets, but are available from the corresponding author in a redacted form on reasonable request.

## Abbreviations

AUC: Area Under Curve
BSA: Bovine Serum Albumin
CV%: Percent Coefficient of Variation
DPR: Differential Positive Ratio
ELISA: Enzyme-Linked Immunosorbent Assay
LPS: Lipopolysaccharide
MES: 2-(N-morpholino)ethanesulfonic acid
MFI: Median Fluorescence Intensity
MFIA: Multiplexed Fluorescent ImmunoAssay
PRRSV: Porcine Reproductive and Respiratory Syndrome Virus
QC: Quality Control
ROC: Receiver Operating Curve
R-PE: R-PhycoErythrin
RT: Room Temperature
S/P%: Percent Sample-to-Positive ratio
SPF system: Specific Pathogen Free system
