# Supplementary material for: Development and evaluation of a bead-based Multiplexed Fluorescent ImmunoAssay (MFIA) for detection of antibodies to Salmonella enterica serogroup B and C1 in pigs
Source: BMC Vet Res. 2022 Jul 5;18:259. doi: 10.1186/s12917-022-03362-w (PMC9254579; doi:10.1186/s12917-022-03362-w)

**Supplementary Fig. 1**

Test of linearity in MFIA when titrating *Salmonella* serogroup B positive serum samples. Four serogroup B positive sera (A-D) with high calculated S/P% values were serially diluted in negative serum. The serum dilutions as well as the negative serum were analysed on two separate days. Figure A-D show mean median fluorescent intensities (MFI) ± STD for serogroup B responses, as well as a linear trend line and R^2^ for the linearity. The higher the MFI, the more specific antibodies are present in the sample. % serum sample represents the percentage of positive sample in negative serum.


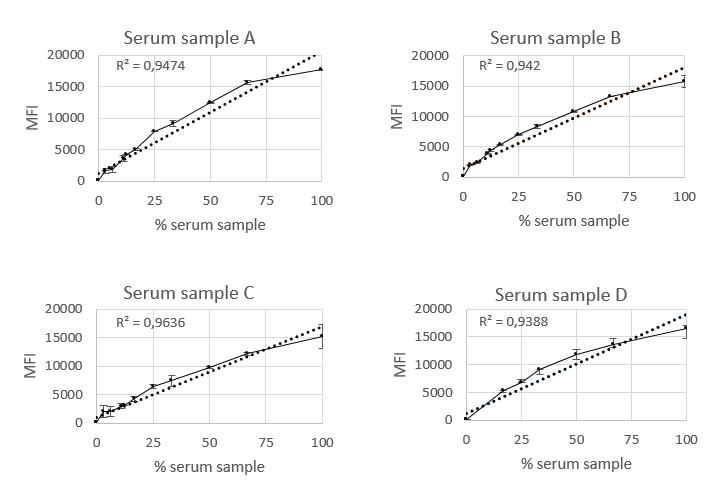

Supplement: Supplementary file 1 — Additional file 1: Supplementary Fig. 1. Test of linearity in MFIA when titrating Salmonella serogroup B positive serum samples. Four serogroup B positive sera (A-D) with high calculated S/P% values were serially diluted in negative serum. The serum dilutions as well as the negative serum were analysed on two separate days. Figure A-D show mean median fluorescent intensities (MFI) ± STD for serogroup B responses, as well as a linear trend line and R2 for the linearity. The higher the MFI, the more specific antibodies are present in the sample. % serum sample represents the percentage of positive sample in negative serum. [file 12917_2022_3362_MOESM1_ESM.docx]
